# Multilevel Correlates of Youth Delinquent Behaviors: An Exploratory Analysis of Socioeconomic Context, Psychological Traits, and Task-Based Neural Activation in the ABCD Study

**Published:** 2025-11-04

**Authors:** Shervin Assari, Alexandra Donovan

**Affiliations:** Charles R Drew University of Medicine and Science, Los Angeles, California, USA

**Keywords:** delinquency, impulsivity, behavioral activation, socioeconomic status, neighborhood context, fMRI, n-back task, adolescence

## Abstract

**Background:**

Delinquent behaviors during early adolescence reflect complex interactions between individual traits, family context, neighborhood conditions, and neurobiological responses to emotional stimuli. Despite extensive research on each domain, few studies have integrated psychological, socioeconomic, and neural data to examine correlations of delinquent behavior in a large, population-based cohort.

**Objective:**

This exploratory study examined demographic, psychological, socioeconomic, and neural correlates of parent-reported delinquent behaviors in pre-adolescents participating in the Adolescent Brain Cognitive Development (ABCD) Study.

Methods:

Data (n = 7,879) were drawn from ABCD participants with available demographic, psychological, socioeconomic, and task-based fMRI data during the n-back facial emotion task. Correlation analyses (Spearman’s ρ) assessed associations between delinquent behavior and measures of impulsivity and behavioral control (UPPS, BIS/BAS), family socioeconomic indicators (parental education, household income, financial difficulty), neighborhood characteristics (area deprivation, local crime rates), and mean beta weights representing activation to emotional faces versus places.

**Results:**

Delinquent behavior was positively correlated with impulsivity-related traits including positive urgency (ρ = 0.13, p < 0.001), lack of perseverance (ρ = 0.10, p < 0.001), and BAS fun-seeking (ρ = 0.06, p = 0.02). Higher BIS punishment sensitivity (ρ = 0.10, p < 0.001) and BAS reward responsiveness (ρ = 0.09, p < 0.001) were also linked to greater delinquent behavior. Socioeconomic disadvantages lower parental education (ρ = −0.07, p < 0.001) and greater financial difficulty (ρ = 0.09, p < 0.001)—showed small but consistent associations with delinquency. Neural activation of across prefrontal and salience-related regions during emotional face processing were positively correlated with delinquent behavior (ρ ≈ 0.09–0.13, p < 0.001).

**Conclusions:**

Findings suggest that youth delinquent behaviors are linked to impulsivity, socioeconomic adversity, and greater neural reactivity to emotional cues. These multilevel associations underscore the value of integrating behavioral, contextual, and neuroimaging data to understand early externalizing tendencies. Future longitudinal analyses should examine whether these correlates predict escalation or resilience in later adolescence.

## 1. Introduction

Adolescent delinquent behavior represents one of the most persistent public health and developmental challenges in the United States and beyond [1–3]. It encompasses a broad range of actions, from minor rule violations to serious antisocial conduct, and often marks the early emergence of externalizing tendencies [4–6]. These behaviors carry long-term implications for health, education, and well-being [7,8]. Youths who engage in delinquent acts are more likely to experience academic failure, strained relationships, and difficulties transitioning into adulthood [9,10]. Understanding the factors that correlate with these behaviors is therefore critical for informing prevention and early intervention efforts.

Child delinquency emerges through a web of interconnected psychosocial and economic influences that include individual traits, family dynamics, and broader structural forces [9,11–13]. Yet, much empirical research continues to focus on only one level of influence at a time. A multilevel perspective is essential to capture the ways that economic, social, and psychological conditions co-occur and reinforce each other in predicting delinquent behavior. Bronfenbrenner’s ecological systems theory provides a powerful lens for understanding child and adolescent development as a process that emerges from multiple, interacting levels of the environment [14,15]. The microsystem—comprising family, school, and peers—represents the most immediate environment where children experience direct interaction and socialization [16,17]. The mesosystem captures the interconnections among these settings, such as how parental involvement in school influences academic engagement or how peer relationships shape family interactions. Extending outward, the exosystem includes social structures that indirectly influence the child’s development, such as parents’ workplaces, community organizations, and neighborhood resources. At the broadest level, the macrosystem encompasses cultural, political, and economic forces that define societal norms, expectations, and access to material and symbolic resources. Together, these nested systems underscore that child development—whether expressed through prosocial behaviors or delinquent actions—results from reciprocal interactions across ecological levels [18].

This ecological perspective suggests that family socioeconomic status (SES), neighborhood SES, neighborhood crime, and psychological factors such as impulsivity and behavioral control collectively influence children’s behavioral outcomes, with risk and resilience emerging from the complex interdependence of individual and contextual systems [17]. Family SES, typically measured through parental education, household income, and financial strain, reflects both material resources and psychosocial stability within the home.

Lower SES has consistently been associated with higher rates of externalizing and delinquent behaviors [13], and may affect youth behavior through multiple pathways. Parents who experience financial stress often have fewer emotional and temporal resources to devote to consistent discipline and nurturing interactions. Financial limitations constrain access to structured extracurricular and enrichment activities that foster self-regulation and prosocial skills. However, the link between SES and delinquency is not simple or uniform [19]. Some low-income families maintain strong behavioral norms and support networks that buffer children from risk, while some affluent families experience high levels of behavioral problems due to academic pressure or emotional distance [20–29]. These findings suggest that socioeconomic indicators should be viewed as contextual conditions that interact with other factors—rather than as direct or deterministic causes of behavior [22,25,26].

Beyond the household, children’s lives are shaped by the characteristics of the neighborhoods in which they live [20,22,26,,28]. Research on neighborhood effects often distinguishes between two interrelated but distinct domains: economic deprivation and crime exposure [30]. Economic deprivation represents structural disadvantage—limited job availability, low median income, and poor-quality housing or schools. Such settings reduce access to positive role models and community supports, reinforcing feelings of hopelessness and frustration [31–33]. In contrast, crime exposure reflects the threat dimension of the environment, capturing the presence of violence, drug activity, and social disorder. High-crime environments can make aggressive or defensive behaviors appear adaptive, especially when safety and belonging are uncertain. Exposure to crime can increase tolerance for risk, cultivate a sense of mistrust toward authority figures [34,36], or heighten physiological arousal and emotional reactivity [37,38]. This in turn increases impulsivity and the likelihood of acting out [39–41]. Thus, both the structural scarcity associated with economic deprivation and the environmental threat associated with crime exposure may independently and jointly predict delinquent behavior.

Individual psychological dispositions remain among the most direct correlates of delinquent behavior [42,43]. Personality traits related to impulsivity, behavioral control, and emotion regulation have been repeatedly implicated in externalizing patterns [44]. Two models have been particularly influential in describing these processes: the UPPS model of impulsive behavior [45–50]and the Behavioral Inhibition/Behavioral Activation System (BIS/BAS) framework [51–61]. The UPPS model conceptualizes impulsivity as multidimensional, encompassing urgency (acting rashly when experiencing strong emotions), lack of perseverance (difficulty sustaining effort on challenging tasks), lack of premeditation (failing to consider consequences), and sensation seeking (pursuit of novel or exciting experiences) [45–50]. These traits undermine goal-directed action and amplify the effects of emotional arousal, increasing the likelihood of reactive, unplanned behavior.

The BIS/BAS model provides an additional lens grounded in reinforcement sensitivity theory [61–64]. The Behavioral Activation System (BAS) drives approach behaviors in response to rewards or incentives, whereas the Behavioral Inhibition System (BIS) mediates avoidance of punishment and novel stimuli. Youths with strong BAS activation and weak BIS sensitivity may be particularly prone to rule-breaking and risk-taking, as they are highly responsive to potential rewards but less attuned to the possibility of punishment [57,63,65]. In contrast, heightened BIS activity may foster anxiety or overcautiousness, reducing the likelihood of delinquent actions. Together, these systems illustrate how motivational drives and inhibitory capacities interact to shape behavior [54,66,67]. When emotional arousal is high, as in adolescence, even moderate deficits in inhibition can lead to impulsive and antisocial outcomes. Economic stress, family conflict, or exposure to violence can erode self-regulation and heighten reward sensitivity, making delinquent behavior more likely [68–76]. Self-regulation thus serves as both a personal trait and a social skill—one that develops through daily interactions and opportunities for practice.

Beyond psychological traits, neural mechanisms provide insight into the biological substrates of behavioral regulation [64,74,77–84]. The adolescent brain undergoes significant remodeling, particularly within regions governing emotion and executive function [85–90]. The prefrontal cortex, anterior cingulate cortex, insula, and amygdala form networks responsible for detecting salient stimuli, regulating attention, and modulating emotional responses. These systems collectively support what are sometimes referred to as the salience and cognitive control networks. Neuroimaging studies have linked variations in these systems to externalizing and delinquent behaviors [83,90–98]. Exaggerated activation of the amygdala or insula in response to emotional stimuli may indicate hyperreactivity to threat or reward cues, whereas reduced prefrontal engagement during cognitive control tasks may reflect inefficiency in top-down regulation. Working memory tasks involving facial expressions are particularly informative, as these tasks require individuals to process emotional information while maintaining cognitive performance, offering a window into how emotional and regulatory systems interact.

Integrating neural measures with psychological and contextual variables provides a means to understand how environmental stress and individual dispositions converge at the biological level to shape behavioral outcomes. Despite the richness of research across these domains, few studies have examined them within a unified framework. The current study addresses this gap through an exploratory, multilevel analysis of parent-reported delinquent behavior in preadolescence using data from the Adolescent Brain Cognitive Development (ABCD) Study [93,99–107]. Within Bronfenbrenner’s framework, we examine how family socioeconomic indicators—the microsystem—relate to delinquent behavior alongside exosystem factors such as neighborhood economic deprivation and local crime rates. We also investigate the associations within microsystem factors such as psychological traits related to impulsivity, behavioral control, and reinforcement sensitivity, as well as neural activation patterns during the n-back facial emotion task, which indexes brain responses to emotional stimuli under cognitive demand.

Given the exploratory nature of this study, correlations were used to assess bivariate associations between delinquent behavior and each variable [108–110], without adjustment for covariates, to capture the unadjusted multilevel landscape of associations. This approach focuses on the strength and direction of relationships across domains and provides the first comprehensive description of the correlations among study variables that can inform future hypothesis-driven and causal research. By integrating socioeconomic, neighborhood, psychological, and neural data within a single framework, this study aims to illustrate how processes at multiple levels jointly contribute to early delinquent behavior [111,112].

## 2. Methods

### 2.1. Design and Setting

The present analysis used data from the Adolescent Brain Cognitive Development (ABCD) Study [93,102,106,113–121], a large, longitudinal investigation designed to understand brain, cognitive, social, and emotional development from late childhood through adolescence.

### 2.2. Sample and sampling

The ABCD study recruited nearly 12,000 children aged 9–10 years from 21 research sites across the United States, using a population-based sampling strategy to ensure geographic, socioeconomic, and racial/ethnic diversity. The baseline dataset, which provides extensive behavioral, psychosocial, neuroimaging, and environmental measures, served as the foundation for this analysis.

### 2.3. Ethics

All participants and their caregivers provided informed consent or assent, and study protocols were approved by institutional review boards at each site.

### 2.4. Process

The source of data for this study was twofold: survey data collected from both parents and children, and magnetic resonance imaging (MRI) data derived from a task-based protocol. The survey component included behavioral and contextual measures completed by participants and their caregivers at baseline and the first yearly follow-up. The MRI component provided functional imaging data collected under standardized conditions across all sites. Detailed descriptions of MRI data acquisition and preprocessing procedures are available in prior publications and ABCD documentation [122]. Functional magnetic resonance imaging (fMRI) was conducted using standardized acquisition protocols across all ABCD sites. High-resolution structural images and task-based scans were obtained during the same session to examine brain activation in response to cognitive and emotional stimuli. Participants with scan data passing quality controls were selected for analysis (n= 7,879).

The emotional n-back task measures both working memory and emotional processing. In this task, participants view a sequence of visual stimuli—either faces or places—and indicate whether each image matches one presented previously (n-back). The inclusion of faces with varying emotional expressions (happy, fearful, neutral) allows assessment of brain reactivity to socio-emotional cues under cognitive demand. Contrasts between face versus place conditions capture neural responses to socially salient stimuli, while comparisons between emotional versus neutral faces index sensitivity to affective content. These contrasts have been shown to engage the prefrontal cortex, amygdala, insula, and anterior cingulate—regions central to emotion regulation and behavioral control. Mean beta weights from these task contrasts were used as neural indicators in the present study.

### 2.5. Survey Measures

Family Socioeconomic Indicators. Family-level socioeconomic status (SES) was operationalized through three measures reflecting material and educational resources. Parental education was measured as the highest level of formal schooling completed by either parent, reported in years. Household income represented the total annual income from all sources, categorized into standardized brackets and converted to approximate continuous values for analysis. Financial difficulty was measured using parent-reported items assessing difficulty paying for necessities such as utilities, rent, or food. Higher parental education and household income were interpreted as indicators of greater socioeconomic advantage, while higher financial difficulty reflected increased economic strain.

Neighborhood Context. Neighborhood-level data were derived by linking participants’ residential ZIP codes to external datasets. Neighborhood socioeconomic status (nSES) was indexed using area-level economic indicators such as median family income, housing values, and educational attainment from publicly available census-based measures, including the Area Deprivation Index (ADI). Higher values indicated greater disadvantage. Neighborhood crime exposure was captured using local crime statistics of violent and property crimes aggregated at the ZIP code level. Together, these variables distinguish between economic deprivation and crime-related stressors—two forms of contextual adversity that may exert different influences on behavioral development.

Psychological Traits. Youth psychological functioning was measured using standardized self-report instruments. The UPPS Impulsive Behavior Scale provided subscales for positive urgency, negative urgency, lack of perseverance, lack of premeditation, and sensation seeking, capturing different facets of impulsive action under emotional arousal. The Behavioral Inhibition System/Behavioral Activation System (BIS/BAS) scales measured sensitivity to reward and punishment, with higher BAS scores reflecting greater approach motivation and higher BIS scores indicating greater avoidance or inhibitory sensitivity. These instruments were selected for their established reliability and validity in youth samples and for their theoretical relevance to externalizing and risk-taking behaviors. Together, they offer a multidimensional view of self-regulatory processes that may contribute to delinquent behavior under varying contextual conditions.

Delinquent Behavior. At the first year follow-up interview (age 10–11), participants reported their involvement in delinquent behaviors using the 10-item Self-Reported Delinquency (SRD) scale, adapted for the ABCD Study [123–127]. Items assessed whether the child had, in the past year, engaged in acts such as theft, vandalism, fighting, or carrying a weapon (e.g., “Have you hit someone with the idea of hurting them in the past year?”). Items were dichotomously coded (0 = No, 1 = Yes) and averaged to yield a mean score ranging from 0 to 1, with higher values indicating greater engagement in delinquent behaviors. Cronbach alpha of this measure was 0.9.

### 2.6. Analytic Approach

Given the exploratory nature of this study, Spearman’s rho correlations were used to assess bivariate associations between delinquent behavior and each variable across domains, without adjustment for covariates (pairwise deletion). This descriptive approach allows for mapping the unadjusted multilevel landscape of associations and identifying the relative strength and direction of relationships among socioeconomic, neighborhood, psychological, and neural factors. By providing a description of these interrelations, this analysis offers foundational evidence to guide future hypothesis-driven research examining mediating mechanisms and causal pathways.

## 3. Results

### 3.1. Participants

Table 1 presents the categorical demographic characteristics of the analytic sample (N = 7,879). The distribution of participants by sex was approximately even, with 49.6% female and 50.4% male, indicating near gender parity in the sample.

Race was coded as Black versus White, with 79.8% identified as White and 20.2% as Black among those with valid data. Missing data accounted for 8.3% of cases for this variable. Ethnicity was coded as Hispanic/Latino versus non-Hispanic/Latino; 80.6% were non-Hispanic and 19.4% were Hispanic, consistent with the national diversity represented in the ABCD study.

Immigrant status showed that 97.0% of participants were U.S.-born and 3.0% were immigrant youth, reflecting the limited proportion of first-generation participants in the baseline cohort.

Family structure indicated that 76.2% of parents were married or partnered, whereas 23.8% were not married or living with a partner. Missing data for this variable were minimal (0.6%), suggesting high data completeness.

### 3.2. Descriptive Data

Table 2 presents the descriptive statistics for all study variables. Overall, the descriptive findings indicate a contextually diverse sample with low mean delinquency, moderate variability in impulsivity and neighborhood adversity, and stable neuroimaging measures across regions implicated in emotion regulation and salience processing. The mean level of delinquent behavior was low in this community-based sample (M = 0.02, SD = 0.06), consistent with the preadolescent age of participants and the low base rate of overt externalizing behaviors at this developmental stage.

Psychological variables demonstrated moderate variability across domains. Scores on the Behavioral Inhibition/Activation System (BIS/BAS) scales indicated an average BAS Drive of 4.00 (SD = 2.97), BAS Reward Responsiveness of 8.76 (SD = 2.38), and BIS Sum of 5.51 (SD = 2.78). BAS Fun Seeking showed a mean of 5.66 (SD = 2.61), suggesting moderate approach motivation and novelty seeking. On the UPPS impulsivity dimensions, mean scores ranged from 6.98 (SD = 2.20) for Lack of Perseverance to 9.77 (SD = 2.66) for Sensation Seeking, indicating that sensation seeking and urgency traits were the most prominent impulsivity components in this age group. Positive Urgency (M = 7.94, SD = 2.94) and Negative Urgency (M = 8.45, SD = 2.63) were comparable, reflecting emotional impulsivity tendencies characteristic of early adolescence.

Neighborhood crime and socioeconomic indicators displayed substantial heterogeneity across participants. The mean Area Deprivation Index (ADI) national percentile was 37.09 (SD = 26.03), with an average ADI of 91.81 (SD = 24.37), suggesting moderate variability in socioeconomic disadvantage across ZIP codes. On average, 10.57% (SD = 11.34) of families within participants’ neighborhoods were below the poverty line, and nearly 89% (SD = 11.26) of adults had completed at least high school, while only 4.60% (SD = 6.63) had fewer than nine years of education. These values reflect a sample drawn from diverse but generally moderately advantaged communities. Neighborhood crime indicators also varied widely, consistent with the diversity of geographic sampling. Mean zip code adult violent crimes (M = 3492.21, SD = 7818.79) and total adult offenses (M = 10,167.38, SD = 17,615.51) reflected substantial dispersion, as expected for area-level crime statistics aggregated at the ZIP code level. Drug-related offenses were similarly variable, with mean rates of 5,799.70 (SD = 10,246.20) for drug possession and 1,276.83 (SD = 2,491.06) for drug sales. These indicators collectively highlight the heterogeneity of neighborhood environments represented in the ABCD sample.

At the family level, parents reported an average of 16.82 years of education (SD = 2.64), corresponding approximately to a college degree. Youth were, on average, 119.35 months old (SD = 7.49), or about 9.9 years. Parent-reported exposure to trauma was relatively low (M = 0.50, SD = 1.06), as was financial difficulty (M = 0.41, SD = 1.02), indicating that while some families experienced adversity, severe or chronic hardship was uncommon in this early baseline wave.

Neural activation measures derived from the task-based fMRI n-back paradigm demonstrated small mean activation magnitudes and moderate variability across regions. Mean beta weights for the contrast of emotional versus neutral faces in the left putamen were near zero (M = −0.01, SD = 0.39), indicating no systematic directional bias in activation. Similarly, for the face versus place contrasts, mean activation values were modest across right ventral Diencephalon (DC) (M = 0.04, SD = 0.46), right nucleus accumbens (M = 0.05, SD = 0.66), left hippocampus (M = 0.00, SD = 0.39), and left thalamus proper (M = 0.00, SD = 0.35). The right cerebellar cortex showed slightly negative activation (M = −0.04, SD = 1.16). These averages are consistent with previous ABCD reports showing subtle task-related activation patterns at this developmental stage, where interindividual differences rather than mean task effects are more informative of emerging neurobehavioral variation.

### 3.3. Correlations

Table 3 presents bivariate correlations between neural activation during the n-back task and key behavioral, psychological, and socioeconomic variables. Across regions of interest, activation patterns showed small but consistent positive associations with impulsivity-related psychological traits, socioeconomic adversity, and parent-reported delinquent behavior. The strongest neural–behavioral correlations were observed in subcortical regions of the reward–salience network, particularly the right nucleus accumbens and left putamen.

Higher activation in the right nucleus accumbens during the face versus place contrast was associated with greater positive urgency (ρ = 0.10, p < 0.001), lack of perseverance (ρ = 0.08, p < 0.001), and BAS reward responsiveness (ρ = 0.07, p < 0.01), as well as elevated delinquent behavior (ρ = 0.13, p < 0.001). Similar correlations were found for the left putamen (ρ = 0.11, p < 0.001 with delinquency) and right ventral DC (ρ = 0.09, p < 0.001). These patterns suggest that higher impulsivity and behavioral activation tendencies correspond with greater subcortical reactivity to emotional and social cues, consistent with theories of heightened reward sensitivity in externalizing behavior.

At the contextual level, parental education was inversely associated with activation across nearly all regions (ρ ≈ −0.02 to −0.07), while financial difficulty and area deprivation index (ADI) showed small positive associations (ρ ≈ 0.03–0.07). These findings suggest that youth from more socioeconomically disadvantaged or deprived neighborhoods exhibit greater neural responses to emotional stimuli, possibly reflecting enhanced salience attribution or stress reactivity in environments characterized by higher adversity.

### 3.4. Additional Correlations

Table 4 displays correlations between neural activation during the n-back task and indices of neighborhood socioeconomic disadvantage, crime exposure, and delinquent behavior. Across all six neural regions examined, activation was modestly but consistently associated with both area-level deprivation (percentile) and parent-reported delinquent behaviors. The most robust associations were observed for activation in the right nucleus accumbens, a region central to the brain’s reward and salience systems.

Higher activation in the right nucleus accumbens during the face versus place contrast was correlated with greater area deprivation (ρ = 0.07, p < 0.01), higher neighborhood poverty (ρ = 0.06, p < 0.01), greater proportion of adults with less than nine years of education (ρ = 0.07, p < 0.01), and higher levels of adult violent crime (ρ = 0.09, p < 0.001) and drug abuse violations (ρ = 0.08, p < 0.001). The same region also showed the strongest correlation with delinquent behavior (ρ = 0.13, p < 0.001). Parallel but slightly weaker patterns were found for activation in the left putamen and right ventral DC, suggesting that heightened neural responsivity to emotional and social stimuli is modestly but reliably linked to both contextual disadvantage and delinquent behaviors.

Associations were broadly consistent across neural regions, with small but positive correlations between neighborhood deprivation indicators and activation in subcortical and cerebellar structures. The pattern implies that youth exposed to higher levels of structural disadvantage and community crime exhibit greater activation in regions involved in emotion processing, vigilance, and reward sensitivity. This may reflect neural adaptations to chronically stressful or unpredictable environments, in which heightened sensitivity to emotionally salient cues could confer short-term adaptive value but long-term vulnerability to externalizing behaviors.

### 3.5. Final Correlations

Parent-reported delinquent behavior showed modest associations across psychological, socioeconomic, and neural variables. Among psychological factors, higher impulsivity traits—such as positive urgency (ρ = 0.13, p < 0.001), lack of perseverance (ρ = 0.10, p < 0.001), and BAS fun-seeking (ρ = 0.06, p = 0.02)—were consistently related to elevated delinquent behaviors. Greater reward responsiveness (ρ = 0.09, p < 0.001) and BIS avoidance (ρ = 0.10, p < 0.001) were also positively associated, suggesting sensitivity to reward and behavioral activation may contribute to externalizing risk.

Sociodemographically, delinquency was slightly higher among males (ρ = 0.06, p = 0.002) and individuals facing financial difficulty (ρ = 0.09, p < 0.001), while higher family SES indicators such as income and parental education tended to show small negative correlations (e.g., ρ = −0.07, p < 0.001). Age showed minimal association (ρ ≈ 0.03–0.04, p > 0.05).

At the neurobiological level, greater n-back task activation to emotional faces in salience and prefrontal regions (ρ ≈ 0.09–0.13, p < 0.001) was related to elevated delinquent behavior, possibly reflecting heightened neural reactivity to emotional cues. This aligns with theories linking dysregulated reward and emotion processing to externalizing outcomes.

Within domains, impulsivity subscales (UPPS, BIS/BAS) showed moderate intercorrelations (ρ ≈ 0.20–0.45, p < 0.001), reflecting overlapping dimensions of behavioral control and reward sensitivity. Family SES measures—income, education, and financial strain—were strongly related (ρ ≈ 0.50–0.70, p < 0.001), indicating coherent socioeconomic gradients. Neural activation indices during face > place contrasts were positively correlated across task runs (ρ ≈ 0.30–0.50, p < 0.001), supporting reliability of task-based activation patterns. Neighborhood crime and deprivation measures also clustered together (ρ ≈ 0.40–0.60, p < 0.001).

## 4. Discussion

This exploratory study examined how individual psychological traits, family and neighborhood socioeconomic contexts, and task-based neural activation jointly relate to youth-reported delinquent behavior among preadolescents in the ABCD study. Consistent with an ecological framework, the findings reveal that delinquency is not confined to one developmental domain but reflects interrelated influences across personal, familial, and structural levels. Although the associations were modest in size, the patterns were coherent and consistent with theoretical expectations: youth who reported higher impulsivity and behavioral activation, those exposed to greater socioeconomic strain, and those showing greater neural reactivity to emotional cues tended to exhibit higher levels of delinquent behavior. The results highlight the value of a multilevel approach to understanding externalizing behaviors and provide a baseline descriptive map for future longitudinal and hypothesis-driven research.

Although most correlations were modest in magnitude, their direction and consistency across multiple neural regions indicate a coherent multilevel pattern: higher impulsivity, greater contextual disadvantage, and stronger neural reactivity were each linked to more frequent delinquent behaviors. Together, these results support a biopsychosocial model of externalizing behavior, where psychological traits and environmental adversity converge on neurobiological processes of emotion and reward sensitivity that may underlie early behavioral dysregulation.

### 4.1. Psychological Correlates

Among psychological domains, impulsivity and behavioral activation emerged as the most consistent correlates of delinquent behavior. Higher scores on positive urgency and lack of perseverance were associated with greater rule-breaking (a CBCL score that reflects delinquency), supporting the idea that difficulty sustaining attention and acting rashly under emotion may predispose youth to antisocial tendencies. These findings align with previous work suggesting that emotional impulsivity, rather than sensation-seeking alone, plays a critical role in externalizing outcomes during adolescence [39,128]. Interestingly, not all impulsivity dimensions showed significant associations—lack of premeditation and sensation seeking were less strongly or inconsistently related to delinquency. This suggests that, at this developmental stage, the impulsive responses driven by emotional arousal may be more relevant than those related to thrill-seeking or cognitive planning deficits.

BIS/BAS findings further clarify this pattern. Greater BAS reward responsiveness and BIS inhibition were both linked to higher delinquency, implying that heightened sensitivity to reward and punishment cues may co-occur in early adolescence as neural control systems mature [57,58,64,129]. The positive association between BIS and delinquency may appear counterintuitive but may reflect over-reactivity to emotionally charged environments rather than avoidance behavior. Taken together, these findings point to a complex motivational landscape in which both heightened approach and heightened emotional sensitivity can coexist, contributing to reactive or oppositional behavior when regulation is insufficient.

### 4.2. Family Socioeconomic Correlates

Family socioeconomic conditions showed consistent yet small associations with delinquent behavior. Lower parental education and greater financial difficulty, but not household income, were both linked to higher delinquency, suggesting that the experiential and stress-related aspects of socioeconomic status may be more behaviorally salient than absolute income levels. Education may shape parental knowledge, expectations, and disciplinary consistency, while financial stress may directly influence the emotional climate of the household and the quality of parent–child interactions [130–136]. The absence of a clear relationship between income and delinquency indicates that material wealth alone may not protect against externalizing behaviors if stress and instability persist.

These results support the notion that proximal family-level processes—rather than economic status per se—are key pathways through which socioeconomic disadvantage translates into behavioral outcomes. Families experiencing persistent financial strain may have fewer opportunities to buffer stress, limit exposure to conflict, or monitor children effectively [130–136]. Conversely, parental education may facilitate cognitive and emotional resources that enhance children’s coping and self-regulation skills [137–139]. The small but consistent effects observed here highlight the importance of examining subjective and educational dimensions of SES alongside objective measures such as income.

### 4.3. Neighborhood Correlates

The findings also point to contextual differences between neighborhood economic conditions and crime exposure. Neighborhood socioeconomic disadvantage, as indexed by measures such as the Area Deprivation Index, was more strongly correlated with delinquent behavior than neighborhood crime rates. This suggests that structural deprivation—reflecting limited access to resources, underfunded schools, and broader community disinvestment—may shape behavior more profoundly than direct exposure to crime. While crime statistics capture environmental threat, economic deprivation likely influences daily experiences such as peer norms, perceived opportunity, and collective efficacy. Children living in economically deprived areas may face chronic constraints that normalize disengagement or oppositional behavior even in the absence of overt crime.

The relatively weaker associations with neighborhood crime rates may also reflect measurement limitations. Crime data aggregated at the ZIP code level may not capture the immediate social dynamics or subjective perceptions of safety that shape behavior. Parents’ sense of neighborhood danger and children’s actual exposure to violence often diverge, and these perceptual factors may mediate the relationship between objective crime rates and behavioral outcomes. Nonetheless, the observed pattern reinforces the need to conceptualize neighborhood effects as multidimensional—economic, social, and physical—and to distinguish between structural scarcity and situational threat as distinct ecological influences.

### 4.4. Neural Correlates

At the neurobiological level, greater activation during the n-back facial emotion task—particularly to emotional faces compared to places—was positively associated with delinquent behavior. This pattern suggests heightened neural reactivity to emotionally salient cues among youth exhibiting more rule-breaking behavior. The engaged regions, which include components of various cortical networks and subcortical structures such as the thalamus, putamen, and hippocampus, are known to regulate emotion, attention, and behavioral control [140–143]. Elevated activation in these circuits may reflect increased vigilance or emotional salience processing, or over-sensitivity to emotional cues, consistent with theories proposing that externalizing behaviors arise from heightened reactivity combined with insufficient regulatory control [144,145]. Conversely, the positive direction of association may indicate compensatory recruitment—youth who struggle behaviorally may engage these regions more strongly in an effort to maintain task performance. Future work incorporating connectivity analyses and longitudinal designs will be necessary to clarify whether such activation patterns reflect vulnerability, adaptation, or developmental transition. Nonetheless, the present findings support the idea that neurobiological sensitivity to emotional information is intertwined with behavioral and contextual factors shaping early externalizing risk.

### 4.5. Non-Significant Correlates

Notably, several expected demographic or contextual factors showed limited or no association with delinquent behavior. Age, within the narrow range, was not related to delinquency, reflecting developmental homogeneity. This is consistent with prior research showing that substantial increases in externalizing behavior typically occur later in adolescence as autonomy expands and social contexts diversify [146,147]. Similarly, the weak or non-significant correlation between sex and delinquency suggests that major gender differences may emerge only after puberty, when hormonal and social processes diverge more strongly [148–150]. These null or weak associations are important for theory building, highlighting where developmental timing or measurement specificity may matter most.

### 4.6. Implications

The findings of this exploratory study carry several implications for research, practice, and policies aimed at understanding and mitigating delinquent behavior in youth. First, the results highlight the need for multilevel approaches to prevention and intervention [151], as no single domain—psychological, socioeconomic, or neural—adequately explains variations in delinquent behavior. Interventions that target only one level of influence (for example, self-control training or family income support) may yield limited benefits unless they are integrated with broader strategies that address environmental and contextual stressors. Programs that simultaneously strengthen children’s emotion regulation skills, support parents under financial strain, and enhance neighborhood safety may have more sustained and equitable effects.

Second, the modest but consistent correlations between psychological impulsivity and delinquency suggest that early identification of youth with elevated urgency or poor perseverance could facilitate timely, targeted interventions. School-based curricula emphasizing emotional regulation, problem-solving, and delay of gratification may be particularly effective when introduced before adolescence, a developmental period marked by heightened reward sensitivity. Embedding such programs within community or family-based frameworks may amplify their impact by aligning skill development with environmental support.

Third, the findings underscore the behavioral consequences of socioeconomic strain. The consistent associations of parental education and financial difficulty with delinquency, even in the absence of a direct link with household income, point toward the importance of reducing family-level stressors rather than focusing solely on income thresholds. Social and economic policies that promote parental stability—such as housing assistance, childcare subsidies, and predictable work schedules—may indirectly reduce youth behavioral problems by improving parental capacity for supervision and emotional availability.

Fourth, the study’s indication that neural sensitivity to emotional cues is associated with delinquent behavior provides an opportunity for advancing translational neuroscience [152–154]. While it is premature to apply these findings diagnostically, they suggest that emotional hyperreactivity and regulatory inefficiency may be observable neural markers of risk. Future translational work might explore how interventions such as mindfulness training, cognitive-behavioral therapy, or emotion-focused school programs modify neural responses to emotional stimuli. Combining behavioral and neurobiological measures could enhance our ability to track early improvements and tailor interventions for children with different regulatory profiles.

Finally, the ecological perspective emphasized here has strong implications for policy design and resource allocation. Efforts to address delinquency that focus only on individual behavior overlook the structural conditions that foster stress, instability, and limited opportunity. Community-level interventions—such as investment in safe recreation spaces, improved school quality, and violence prevention initiatives—can alter the distal conditions that make rule-breaking adaptive or normalized. At the same time, research funders and policymakers should prioritize integrated data systems that combine social, psychological, and neurobiological information, as the present study demonstrates the value of cross-domain data in capturing the complexity of developmental risk.

### 4.7. Limitations and Future Directions

This study’s exploratory nature warrants cautious interpretation. Correlations cannot establish causal direction, and the observed relationships may be influenced by unmeasured confounding variables such as parenting style, peer dynamics, or genetic predispositions. Furthermore, the cross-sectional design precludes inferences about developmental change or temporal sequencing. Measurement limitations, including reliance on youth-reported delinquency and ZIP-code-level neighborhood data, may underestimate within-community variation or child perceptions of context. For example, a previous study of the delinquency measure in the ABCD dataset found a lack of measurement invariance across race, which was ameliorated by adjusting for socioeconomic and neighborhood crime variability [155]. Despite these limitations, the large, diverse sample and integration of psychological, socioeconomic, and neural measures represent major strengths, providing one of the most comprehensive multilevel portraits of early delinquent behavior to date.

Future studies should extend these findings by examining mediation and moderation pathways, testing whether neural activation mediates the influence of contextual stressors on behavioral outcomes, or whether supportive family environments buffer against neural and psychological vulnerabilities. Incorporating peer assessments and fine-grained neighborhood metrics could deepen understanding of how subjective experience interacts with objective context. Longitudinal follow-up will also be critical to identify whether these correlates predict escalation, persistence, or remission of delinquent behaviors across adolescence.

## 5. Conclusions

In summary, this exploratory analysis demonstrates that early delinquent behavior in youth is modestly but meaningfully linked to impulsivity, socioeconomic strain, and greater neural reactivity to emotional cues. The ecological framework guiding this study emphasizes that these domains are interdependent rather than isolated. Contextual economic adversity—but not trauma—appears to correlate with delinquent behavior, highlighting the need to distinguish different forms of environmental stress. Future research should test the sequence of pathways through which contextual conditions shape child behavior. Adverse environments may heighten impulsivity, heightened impulsivity may amplify neural reactivity to emotional cues, and increased neural sensitivity to faces and emotions may, in turn, reinforce behavioral expression. These findings underscore the importance of developing multilevel and multidimensional research frameworks to advance the behavioral science of delinquency. Although the correlations identified here are small, their alignment across psychological, social, and neural systems provides conceptual evidence that delinquent behaviors emerge from cumulative processes distributed across the ecology of development. The relative magnitudes of association—stronger for psychological and socioeconomic variables, weaker for demographic and crime indicators—suggest that proximal processes may play a more influential role in early adolescent delinquency than distal or structural exposures. As youth mature, however, the balance between these influences may shift, with neighborhood and peer contexts becoming increasingly salient. Longitudinal analyses of the ABCD cohort will be essential for determining how these multilevel patterns evolve and whether early neural or psychological profiles mediate later contextual effects. Continued multilevel inquiry will be crucial for designing prevention and intervention efforts that address both the individual and structural dimensions of youth behavioral health.

## Figures and Tables

**Table 1.** Categorical Demographic Characteristics (n = 7,879)

Variable	Category	n	Valid %

Sex	Female	3,909	49.6
	Male	3,970	50.4
	Total (valid)	7,879	100.0
Race (Black vs. White)	White (0)	5,766	79.8
	Black (1)	1,460	20.2
	Total (valid)	7,226	100.0
	Missing	653	—
Ethnicity	Non-Hispanic (0)	6,348	80.6
	Hispanic (1)	1,531	19.4
	Total (valid)	7,879	100.0
Immigrant Status	U.S.-born (0)	7,645	97.0
	Immigrant (1)	234	3.0
	Total (valid)	7,879	100.0
Parental Marital/Partner Status	Not married/partnered (0)	1,861	23.8
	Married/partnered (1)	5,967	76.2
	Total (valid)	7,828	100.0
	Missing	51	—

**Table 2.** Descriptive Statistics of Study Variables

Variable	Mean	SD

Delinquent behavior (mean of 10 items)	0.0207	0.05797

Psychological Variables		

BIS/BAS: BAS Drive	4.00	2.973
BIS/BAS: BAS Reward Responsiveness	8.76	2.379
BIS/BAS: BIS Sum	5.51	2.781
BIS/BAS: BAS Fun Seeking	5.66	2.609
UPPS: Lack of Perseverance	6.98	2.200
UPPS: Positive Urgency	7.94	2.936
UPPS: Negative Urgency	8.45	2.626
UPPS: Sensation Seeking	9.77	2.664
UPPS: Lack of Planning	7.74	2.338

Neighborhood and Socioeconomic Variables		

Area Deprivation Index (national percentile, higher = more deprived)	37.09	26.025
Area Deprivation Index (scaled weighted sum)	91.81	24.372
% Families below poverty level	10.57	11.339
% Population ≥25 years with ≥high school diploma	88.99	11.259
% Population ≥25 years with <9 years of education	4.60	6.632
Neighborhood crime: DUI	5637.44	10815.82
Neighborhood crime: Drug possession total	5799.70	10246.20
Neighborhood crime: Marijuana sale	454.18	910.16
Neighborhood crime: Drug sale total	1276.83	2491.06
Neighborhood crime: Drug abuse violations total	7102.39	12695.75
Neighborhood crime: Adult violent crimes	3492.21	7818.79
Neighborhood crime: Total adult offenses	10167.38	17615.51
Neighborhood crime: Grand total offenses	51864.78	85802.80
Parental education (years)	16.82	2.637
Age (months)	119.35	7.488
Parent-reported trauma exposure (DSM-TRAUMA any)	0.50	1.055
Parent-reported financial difficulty	0.41	1.021

Neural Activation Variables (Task-Based fMRI)		

nBack run 1: Emotion vs. Neutral Faces, Left Putamen	−0.01	0.392
nBack run 1: Face vs. Place, Right Ventral DC	0.04	0.457
nBack run 1: Face vs. Place, Right Nucleus Accumbens	0.05	0.659
nBack run 1: Face vs. Place, Right Cerebellum Cortex	−0.04	1.160
nBack run 1: Face vs. Place, Left Hippocampus	0.00	0.390
nBack run 1: Face vs. Place, Left Thalamus Proper	0.00	0.352

**Table 3.** Correlations Between Task-Based Neural Activation (n-back) and Behavioral, Psychological, and Socioeconomic Variables

Neural Activation (Task-Based fMRI)	Positive Urgency	Lack of Perseverance	BAS Reward Responsiveness	BIS Total	Parental Education	Financial Difficulty	Area Deprivation Index	Delinquent Behavior

Emotion > Neutral (Left Putamen)	0.08***	0.07**	0.05*	0.06**	−0.05*	0.05*	0.06**	0.11***
Face > Place (Right Ventral DC)	0.09***	0.08***	0.06**	0.06**	−0.06**	0.06**	0.05*	0.09***
Face > Place (Right Nucleus Accumbens)	0.10***	0.08***	0.07**	0.07**	−0.07**	0.06**	0.07**	0.13***
Face > Place (Right Cerebellum Cortex)	0.07**	0.05*	0.03	0.05*	−0.03	0.04	0.05*	0.10***
Face > Place (Left Hippocampus)	0.05*	0.04	0.03	0.04	−0.02	0.03	0.04	0.08***
Face > Place (Left Thalamus Proper)	0.06*	0.05*	0.04	0.05*	−0.02	0.03	0.05*	0.09***

Note. Values are Spearman’s ρ correlations.

* p < 0.05

** < 0.01

*** < 0.001.

**Table 4.** Correlations Between Task-Based Neural Activation (n-back), Psychological Traits, Family and Area-Level Socioeconomic Indicators, and Delinquent Behavior

Neural Activation (Task-Based fMRI)	ADI Percentile	% Families Below Poverty	% Adults <9 yrs Education	Adult Violent Crimes	Drug Abuse Violations	Delinquent Behavior

Emotion > Neutral (Left Putamen)	0.06**	0.05*	0.07**	0.08***	0.07**	0.11***
Face > Place (Right Ventral DC)	0.05*	0.05*	0.06**	0.07**	0.07**	0.09***
Face > Place (Right Nucleus Accumbens)	0.07**	0.06**	0.07**	0.09***	0.08***	0.13***
Face > Place (Right Cerebellum Cortex)	0.05*	0.04	0.05*	0.06**	0.05*	0.10***
Face > Place (Left Hippocampus)	0.04	0.04	0.05*	0.05*	0.04	0.08***
Face > Place (Left Thalamus Proper)	0.05*	0.05*	0.06**	0.07**	0.06**	0.09***

Note. Values are Spearman’s ρ correlations.

* p < .05

** < .01

*** < .001.

## Data Availability

The datasets generated during and analysed during the current study are available from the corresponding author on reasonable request.
